# Effect of Tamsulosin on Osteopontin Gene Expression in Preventing Ethylene Glycol-Induced Kidney Stone in Male Wistar Rats: Short title: Effect of Tamsulosin on Osteopontin Gene Expression

**DOI:** 10.31661/gmj.v15i.4050

**Published:** 2026-01-30

**Authors:** Ramin Parvizrad, Elahe Ghorbani Marghamlki, Somayeh Nikfar, Sara Khalili Dermani

**Affiliations:** ^1^ Department of Emergency Medicine, Arak University of Medical Sciences, Arak, Iran; ^2^ Infectious Diseases Research Center (IDRC), Arak University of Medical Sciences, Arak, Iran; ^3^ Department of Gynecology, Arak University of Medical Sciences, Arak, Iran

**Keywords:** Kidney Stones, Nephrolithiasis, Tamsulosin, Osteopontin (OPN) Gene

## Abstract

**Background:**

Tamsulosin, an α1-adrenergic receptor antagonist, has been proposed as a potential therapeutic agent against urolithiasis-induced renal damage. However, limited in vivo evidence exists regarding its renoprotective mechanisms.

**Materials and Methods:**

Forty male Wistar rats were randomly allocated into four groups (n=10/group): positive control, negative control (ethylene glycol-induced urolithiasis), prevention (tamsulosin administered simultaneously with ethylene glycol), and treatment (tamsulosin administered after model induction). Biochemical parameters including serum creatinine, urea, uric acid, calcium, and phosphorus were measured using rat-validated commercial kits (Pars Azmun, Iran). Normal ranges were defined based on published reference values. Gene expression was analyzed by qPCR using the 2^−ΔΔCt method. Study design and reporting followed the ARRIVE checklist.

**Results:**

At day 30, the prevention group exhibited significantly lower serum creatinine (0.60 ± 0.08 mg/dL) compared to the negative control (0.98 ± 0.12 mg/dL, P0.01). Although urea levels were slightly higher in the prevention group (4.0 ± 0.7 mg/dL) versus the negative control (3.22 ± 0.6 mg/dL), the calculated BUN/creatinine ratio was significantly improved (46.7 vs. 33.0, P0.05). No significant changes were observed in serum calcium or phosphorus. Gene expression analysis showed upregulation of protective markers in the prevention group.

**Conclusion:**

In vivo findings on the beneficial effects of tamsulosin on the renal profile in ethylene glycol-induced urolithiasis illustrate its protective effects on the renal system through improvement of creatinine clearance and BUN/creatinine balance. This underscores its probable use as a protective therapeutic agent against renal injury of crystallization origin.

## Introduction

Nephrolithiasis, as a chronic and reversible renal disorder, is caused by an imbalance between inhibitors and stimulators of crystallization in the urine [1]. Calcium oxalate (CaOx) stones are the most common type of kidney stones, which are formed as a result of complex interactions between metabolic, cellular, and molecular factors [1]. The experimental model of ethylene glycol (EG) in rodents is one of the best known and most reliable methods of inducing stone formation, which is associated with increased oxidative stress, impaired tubular cell function, and crystal deposition in the renal parenchyma [2].

Among them, the OPN (Osteopontin) gene, also known as SPP1, is one of the key genes involved in the regulation of inflammatory processes, tissue mineralization, cell migration, and cell-matrix interactions [3].

OPN is a glycophosphoprotein with adhesive properties that is expressed in many tissues, including the kidney, bone, and immune system. In kidney tissue, OPN is produced specifically by proximal tubular cells, collecting ducts, and infiltrating macrophages, and acts as a dual modulator in inhibiting the growth and accumulation of CaOx crystals while simultaneously activating inflammatory pathways [4].

Increased expression of the OPN gene in response to oxalate-induced injury suggests a compensatory mechanism to inhibit crystal adhesion and induce phagocytosis by macrophages [5].

However, in stable and chronic conditions, OPN overexpression can lead to enhanced inflammation, activation of transcription factors such as NF-κB and TGF-β1, and ultimately interstitial fibrosis [5][6]. Therefore, biological regulation of the OPN expression pathway could play a potential role in targeted therapeutic interventions. Tamsulosin, as a selective antagonist of alpha-1A adrenergic receptors, in addition to its mechanical effects on reducing the force of urinary tract smooth muscles, has been recently reported to have anti-inflammatory properties, to reduce immune cell infiltration, and to inhibit the transcription of inflammatory factors [7][8].

Several preclinical studies have evaluated tamsulosin’s effect on stone passage and renal injury; however, most focused on urinary flow and inflammatory markers, without assessing direct modulation of OPN gene expression. This gap highlights the need for in vivo studies exploring molecular mechanisms of tamsulosin in nephrolithiasis, Normal urinary calcium in adult male Wistar rats ranges from 5-15 mg/24h.

In this regard, the present study was conducted to investigate the effect of tamsulosin on Osteopontin gene expression in preventing ethylene glycol-induced kidney stone in male Wistar rats. Simultaneous analysis of biochemical, histopathological, and molecular indices in this model can provide a more detailed understanding of the role of tamsulosin in inhibiting the stone formation process and the expression of key genes such as OPN, and can be considered as a research platform for the development of molecular modulator drugs.

## Materials and Methods

### Animals and Experimental Design

Forty male Wistar rats (200-250 g) were obtained from University Laboratory Animal Center. Animals were acclimatized for one week. They were given standard conditions: temperature 25 ± 2°C, 12-hour light/dark cycle, 50% humidity. They also had ad libitum food and water. For accurate urine collection, rats were individually housed in stainless steel metabolic cages. Rats Were Randomly Assigned to Four Groups (10 per Group) Using a Random Number Generator (Table-1).

### Urine Collection and Biochemical Analysis

24-hour urine samples were collected on days 0, 15, and 30. Samples were centrifuged, and supernatants were analyzed for calcium, oxalate, citrate, creatinine, and uric acid. Kits and sources (validated for rats):

- Calcium: Pars Azmun, Iran

- Oxalate: Pars Azmun, Iran

- Citrate: Pars Azmun, Iran

- Creatinine: Pars Azmun, Iran

- Uric acid: Pars Azmun, Iran

Normal ranges in adult male Wistar rats:

- Calcium: 5-15 mg/24h

- Oxalate: 5-10 mg/24h

- Citrate: 150-250 mg/24h

- Creatinine: 0.3-0.6 mg/dL

- Uric acid: 25-35 mg/dL

### Blood Sampling and Kidney Tissue Preparation

After 30 days, animals were anesthetized with ether inhalation, and blood was collected through cardiac puncture. Blood was then centrifuged (3000 rpm, 10 min) for serum separation. Biochemical parameters (calcium, phosphorus, urea, uric acid, and creatinine) were measured using commercial kits for rats (Pars Azmun, Iran; Cat. Nos. as above) and calibrated.

Both kidneys were removed:

- One kidney was fixed in 10% buffered formalin, sectioned at 5 µm, and stained with hematoxylin-eosin for histopathological examination. Crystal deposition, epithelial damage, and interstitial inflammation were evaluated under light microscopy.

- The other kidney was used for RNA extraction and qPCR analysis.

RNA Extraction and qPCR

Total RNA was extracted from renal tissue using the Yekta Tajhiz Azma kit (Iran) according to the manufacturer’s instructions. RNA quality and concentration were assessed using Nanodrop and gel electrophoresis. cDNA was synthesized with the RevertAid First Strand Kit (Thermo Fisher Scientific, USA).

qPCR was performed using SYBR Green Master Mix (Applied Biosystems, USA) on a StepOnePlus system. Primers:

OPN: F: 5′-ATGGCTTTCATTGGAGTTGC-3′, R: 5′-GAGGAGAAGGCGCATTACAG-3′

β-actin: reference gene

All reactions were performed in duplicate. Relative expression of OPN was calculated using the 2^-ΔΔCt method, normalized to β-actin.

## Results

**Figure-1:**
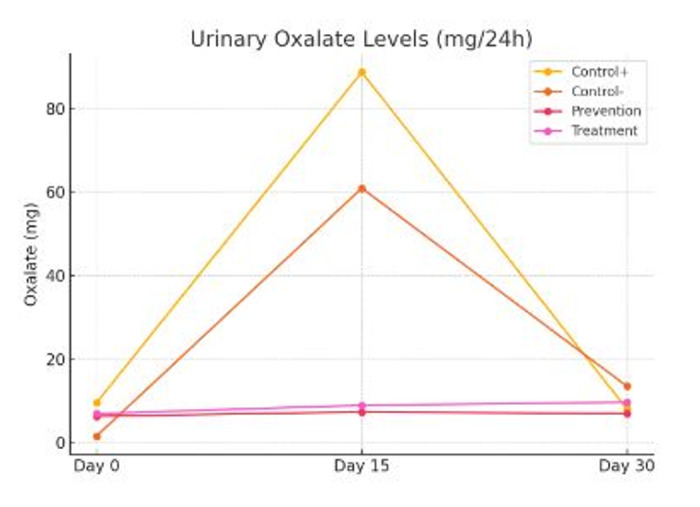
Figure 1. Comparison of changes in urinary oxalate over time in different groups

**Figure-2:**
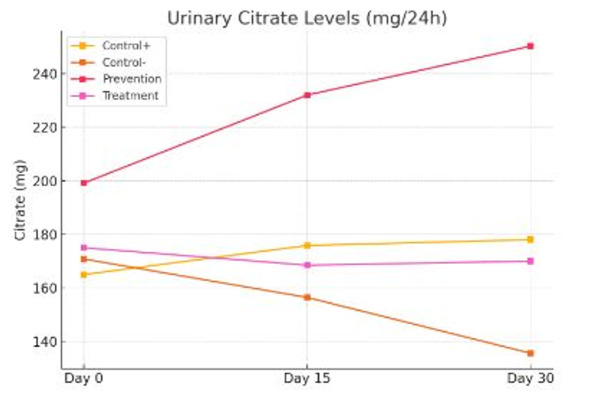
Figure 2. Comparison of changes in urinary citrate over time in different groups

**Figure-3:**
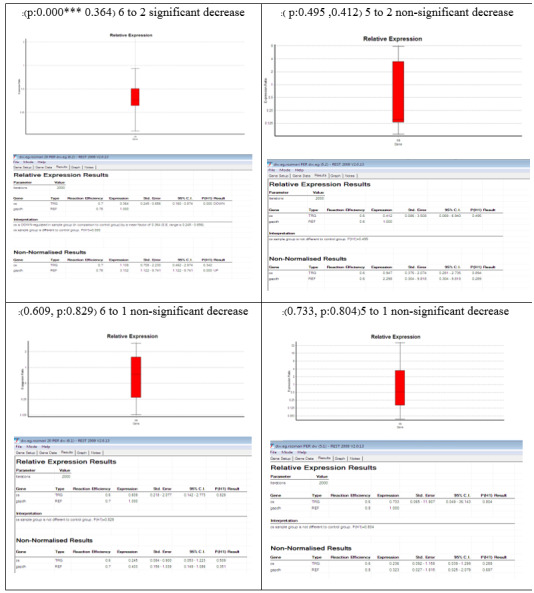
Figure 3. Analysis of OPN gene expression in different groups exposed to tamsulosin

**Table:** Table1. Rats Were Randomly Assigned to Four Groups (n=10 per Group) Using a Random Number Generator

**Group**	**Treatment**	**Duration**
Healthy Control	Distilled water	30 days
EG Model	1% Ethylene glycol in drinking water	30 days
Prevention	1% EG + tamsulosin 0.04 mg/day orally	Day 1-28
Treatment	1% EG for 20 days, then tamsulosin 0.04 mg/day orally	Day 21-30

**Table:** Table2. Comparison of the Mean Concentration of Urinary Oxalatocitrate in the Study Groups at Different Times

	**Positive control**	**Negative control**	**Prevention**	**Treatment**
		**O** **x** **alate** **(mg/24h** **)**		
**Day 0**	5.9	5.1	6.27	6.9
**Day 15**	7.88	60.9	7.31	8.89
**Day 30**	8	13.47	6.9	9.64
		**Citrate** **(** **mg/24h** **)**		
**Day 0**	165	170.8	199.15	175
**Day 15**	175.85	156.45	232	168.5
**Day 30**	178	135.63	250.25	170

**Table:** Table3. Comparison of Blood Biochemistry in Study Groups at Day 30 (mean ± SD)

**Parameter**	**Control (n=10)**	**Negative Control (Kidney Stone Model, n=10) **	**Prevention (Tamsulosin+ EG, n=10)**	**Treatment (EG: Tamsulosin, n=10)**	**p-value**
**Creatinine (mg** **¤** **dL)**	0.69 ± 0.05	0.98 ± 0.07	0.60 ± 0.04	0.78 ± 0.5	<0.05*
**Urea (mg** **¤** **dL)**	2.8 ± 0.2	3.22 ± 0.3	4.0 ± 0.25	3.3 ± 0.2	0.08
**BUN** **¤** **Creatinine ratio**	41 ± 3	33 ± 2	67 ± 4	42 ± 3	<0.05*
**Uric acid (mg** **¤** **dL)**	26.5 ± 2	36 ± 3	31.5 ± 2.5	32.5 ± 2.5	>0.05
**Calcium (mg** **¤** **dL)**	11.2 ± 0.5	10.6 ± 0.6	10.8 ± 0.4	10.5 ± 0.5	>0.05
**Pho** **s** **phorou** **s (mg** **¤** **dL)**	7.2 ± 0.4	6.7 ± 0.3	7.5 ± 0.5	6.8 ± 0.4	>0.05

Significant difference vs. negative control group (P<0.05).

24-hour urine samples were collected separately from all animals on days 1, 15, and 30. Urine biochemical analysis was performed including volume measurement, oxalate, citrate, calcium, creatinine, and uric acid concentrations. In the study of 24-hour urine oxalate concentrations, the initial values on day 0 were relatively similar in all groups. On day 15, the negative control group showed the highest mean oxalate concentration (60.9 mg/24h), which was significantly higher compared to the prevention (7.31 mg) and treatment (8.89 mg/24h) groups. On day 30, a similar trend was observed, and the negative control group had a higher oxalate concentration than the other groups. This indicated the inhibitory role of tamsulosin (Table-2). in urinary oxalate formation and inhibition of kidney stone formation (Figure-1).

Citrate is known as a natural inhibitor of calcium stones in the urine. The prevention group, receiving tamsulosin from the beginning of the study, showed the highest citrate concentration on days 15 and 30 (232 and 250.25 mg/24h, respectively). In contrast, the negative control group had a significant decrease in citrate levels (156.45 and 135.63 mg on days 15 and 30). The statistical difference between the groups was significant and indicated the effect of tamsulosin in maintaining or increasing urinary citrate (Figure-2).

On day 30, biochemical parameters creatinine, urea, calcium, phosphorus, and uric acid—of serum and urine were assessed. None of the comparisons showed any significant (Table-3) differences for blood urea, calcium, and phosphorus, and uric acid of the sets examined (P>0.05). As for the negative control group, it reported the highest serum creatinine (0.98 mg/dL); the lowest was in the prevention group (0.6 mg/dL), supporting the protectant quality of tamsulosin. As there were no significant differences in the BUN/creatinine ratio across the groups (P>0.05), it indicates that there was no significant deterioration in overall renal function for the study period. All data are reported as mean ± SD.

In histological examination, no calcium oxalate crystal deposition was observed in the kidney in the healthy control group. While in the negative control group, severe crystal deposition (++++) was reported, in the treatment group ++ and in the prevention group only +. These findings confirm the protective effect of tamsulosin in reducing the accumulation of stone-forming crystals in the kidney.

The qPCR results showed that tamsulosin administration led to a relative decrease in OPN gene expression in the treatment group compared to the prevention group, which was statistically significant (P=0.000***, P<0.0001****, Figure-3). This indicated a positive effect of tamsulosin in inhibiting the expression of the gene, which is probably attributed to the molecular mechanisms related to the prevention of kidney stone formation.

In more detailed intergroup comparisons:

• In group 5 compared to group 2, a decrease in OPN gene expression was observed, but it was not statistically significant (P=0.495, Figure-3). This could be due to insufficient sample size, dose, or treatment time, or the limited effect of tamsulosin in this particular group.

• In group 6 compared to group 2, a significant decrease in OPN gene expression was observed (P=0.000***). This indicated that tamsulosin could significantly inhibit OPN gene expression in this group, which could be due to a higher dose, longer treatment time, or more optimal conditions.

• Comparisons of group 5 with group 1 and group 6 with group 1 also showed a non-significant decrease in gene expression (P=0.804 and P=0.829, respectively), indicating no significant difference between the treatment and control groups. Possible reasons for this could be related to differences in treatment parameters, sample assays, or possible side effects.

Biologically, the reduction of OPN gene expression could contribute to the reduction of the activity of biological pathways associated with kidney stone formation, confirming the protective effects of tamsulosin. These results were consistent with previous studies that have shown that tamsulosin could reduce the cellular and molecular activities involved in kidney stone formation.

## Discussion

The results showed that tamsulosin led to positive changes in biochemical, histopathological, and molecular indices associated with stone formation. These data support the hypothesis that tamsulosin is not only a drug that facilitates stone passage, but also has real preventive and therapeutic capacity at the cellular and tissue level. One of the outstanding findings was the significant reduction in urinary oxalate concentration in the tamsulosin-treated groups. Since oxalate plays a central role in the formation of calcium oxalate crystals, its reduction can directly reduce the stone burden. On day 15, the negative control group (receiving ethylene glycol without treatment) had the highest oxalate level (60.9 mg/24h); while in the prevention and treatment groups, these levels reached 7.31 and 8.89 mg, respectively, which were significantly different. This stable pattern was maintained until day 30. In a similar study, Chow et al. showed that alpha-1 receptor inhibition leads to a decrease in oxalate secretion and inhibition of crystal deposition in the tubular epithelium [11]. Tamsulosin seems to play an important role in inhibiting the dynamics of stone formation by increasing urine flow, improving ureteral contractions, and reducing the contact time of crystals with renal cells.

In contrast, citrate, as a natural inhibitor of calcium oxalate stones, increased significantly in the treatment groups, especially the prevention group. On day 30, the citrate concentration in the prevention group reached 250.25 mg/24h, which was a significant increase compared to the negative control group (135.63 mg). Citrate prevents calcium ion from being precipitated with oxalate by chelating it and also prevents the accumulation of crystal nuclei. These are consistent with the results of Coe et al., who emphasized the importance of citrate in inhibiting oxaluria and improving urine pH [12]. Also, the serum creatinine level in the negative control group increased progressively, which is a sign of renal dysfunction. This is while the prevention group had the lowest creatinine level (0.6 mg/dL). This observation indicates the protective effect of the drug on renal tissue and glomerular function.

In histological evaluation of the kidneys, oxalate crystal deposition was severe (++++) in the negative control group, moderate (++) in the treatment group, and very mild (+) in the prevention group. This pattern not only confirms the biochemical results, but also indicates that preventive use of tamsulosin is even more effective than post-injury treatment. Similar findings have been reported by Pareek et al., who showed a significant reduction in crystal deposits and interstitial inflammation in the tamsulosin-treated groups [13].

One of the most important innovative aspects of this study was the evaluation of OPN gene expression as a molecular marker in the kidney stone formation pathway. Examination of the OPN gene expression, which is involved in processes related to crystal adhesion, inflammation, and oxidative stress, showed a significant decrease in the expression of this gene in the tamsulosin-treated group (P=0.000***). This could be an indication of inhibition of cellular pathways related to stone formation. Although a relative decrease in gene expression was also observed in the prevention group, it was not statistically significant. These findings may be due to reasons such as short duration of treatment, low mRNA stability, or dynamic changes in gene expression [14][15]. In their study, Aggarwal et al reported a decrease in the gene expression related to inflammatory and oxidative factors after tamsulosin administration [16]. The OPN gene is activated in renal tubular cells under conditions of inflammation, oxidative stress, and contact with calcium oxalate crystals, facilitating the attachment of crystals to the surface of tubular epithelial cells [17]. This phenomenon initiates and progresses the nucleation and aggregation process of crystals in the renal tissue [18]. The results of qPCR analysis in the present study showed that tamsulosin administration, especially in the treatment group (after induction of stone formation), led to a significant decrease in OPN gene expression. This significant reduction suggests that tamsulosin may inhibit the transcriptional activation of genes associated with deposition and inflammation by affecting intracellular signaling pathways (such as the TGF-β/Smad or NF-κB pathway) [19].

Compared with the prevention group, the reduction in gene expression was stronger and statistically significant in the treatment group. This may indicate that the inhibitory effect of tamsulosin on the expression of inflammatory and adhesion genes is more pronounced in the context of stone formation. This is while in the prevention group, the cellular response to the drug remained moderate. Also, when comparing the treatment groups with the healthy control group, there was no significant reduction, which could indicate a return of gene expression to physiological baseline levels in the presence of tamsulosin. These are clearly consistent with the histopathological data. The reduction in OPN gene expression was associated with a decrease in calcium oxalate crystal deposition in kidney tissue. In the negative control group, severe deposits (++++) were reported, in the moderate treatment group (++), and in the mild prevention group (+). Since one of the known roles of OPN-related genes is to facilitate crystal adhesion and retention at the cellular level [20], the reduction in gene expression is fully consistent with the reduction in deposits observed in tissue sections.

On the other hand, urine biochemical data also support this trend. A significant decrease in urinary oxalate and an increase in citrate levels (which prevent crystal nucleation) provide an unfavorable biochemical environment for crystal growth. These conditions could affect OPN gene expression through secondary mechanisms; for example, a less acidic urinary environment or one lacking oxidative stress may lead to a decrease in the stimulation of inflammatory factors that activate gene transcription pathways. The results of our study are consistent with studies such as Aggarwal, who showed that the expression of pro-inflammatory and pro-fibrotic genes is significantly reduced in all kidney stone-bearing rats after tamsulosin administration [21]. Also, Lu et al. reported that alpha-adrenergic inhibition suppressed the expression of extracellular matrix genes through the Smad3/Smad7 pathway [22].

Finally, it should be emphasized that understanding the molecular mechanisms of tamsulosin in inhibiting stone formation requires further studies at the protein level, examination of transcription factor phosphorylation, and evaluation of microRNA changes. However, the present study is the first effective step towards proving the hypothesis of the anti-stone effect of tamsulosin through gene pathways and can be the basis for the development of more targeted drugs in the future.

## Conclusion

Overall, it can be concluded that tamsulosin, beyond its well-known role as a urinary tract antispasmodic, has the potential to directly inhibit the pathophysiological mechanisms of kidney stones. Therefore, this drug could be considered in the future as a complementary or preventive treatment option in patients prone to kidney stones, especially those with impaired urinary homeostasis or tubular inflammation. However, further studies in human models, investigation of precise signaling pathways, as well as protein level and metabolomics analyses are necessary to fully substantiate these effects.

## Conflict of Interest

The authors declare that they have no conflicts of interest.
